# Washing the guilt away: effects of personal versus vicarious cleansing on guilty feelings and prosocial behavior

**DOI:** 10.3389/fnhum.2014.00097

**Published:** 2014-02-28

**Authors:** Hanyi Xu, Laurent Bègue, Brad J. Bushman

**Affiliations:** ^1^Laboratoire Interuniversitaire de Psychologie (LIP), University of Grenoble 2Grenoble, France; ^2^Department of Psychology, University of LouvainLouvain-la-Neuve, Belgium; ^3^School of Communication and Department of Psychology, The Ohio State UniversityColumbus, OH, USA; ^4^Department of Communication Science, VU University AmsterdamAmsterdam, Netherlands

**Keywords:** guilt, wash, cleanse, embodiment, prosocial behavior, helping

## Abstract

For centuries people have washed away their guilt by washing their hands. Do people need to wash their own hands, or is it enough to watch other people wash their hands? To induce guilt, we had participants write about a past wrong they had committed. Next, they washed their hands, watched a washing-hands video, or watched a typing-hands video. After the study was over, participants could help a Ph.D. student complete her dissertation by taking some questionnaires home and returning them within 3 weeks. Results showed that guilt and helping behavior were lowest among participants who washed their hands, followed by participants who watched a washing-hands video, followed by participants who watched a typing-hands video. Guilt mediated the effects of cleansing on helping. These findings suggest that washing one’s own hands, or even watching someone else wash their hands, can wash away one’s guilt and lead to less helpful behavior.

## Introduction

When Jesus Christ was brought before Pontius Pilate, the Roman governor in Jerusalem at the time, Pilate offered to release a prisoner for the Passover feast, either Jesus Christ or the “notorious prisoner” Barabbas. The Jewish chief priests and elders persuaded the people to ask for the release of Barabbas. When Pilate asked what should be done with Jesus Christ, the multitude said, “Let him be crucified” (Matthew 27:22). When Pilate asked, “Why, what evil hath he done?” they cried out again, “Let him be crucified” (Matthew 27:23). Pilate then “took water, and washed his hands before the multitude, saying, ‘I am innocent of the blood of this just person’” (Matthew 27:24). Likewise, in Shakespeare’s play, Lady Macbeth attempted to wash away her guilt of plotting King Duncan’s murder by compulsively washing her hands.

### Guilt

Guilt is an unpleasant emotional feeling that helps us know we did something wrong (e.g., Baumeister et al., 1994; Ferguson and Stegge, 1998). Although guilt feels bad to the individual, it is actually quite good for society and for close relationships. You would not want to have a boss, a lover, a roommate, or a business partner who had no sense of guilt. Such people are called psychopaths, and they are often a disaster to those around them (see Hare, 1998). Psychopaths exploit and harm others, help themselves at the expense of others, and feel no remorse about those they hurt.

When people feel guilty about something they have done, they often perform prosocial actions to wash away the guilt. For example, in one study (McMillen and Austin, 1971), half the participants were induced to tell a lie to the experimenter. After the study was over, the experimenter said that participants were free to go, but added that if they had extra time they could help him fill in bubble sheets for another study. Participants who had not been induced to lie volunteered to help fill in bubble sheets for 2 min on average, whereas participants who had been induced to lie volunteered to help fill in bubble sheets for 63 min. The lying participants were apparently attempting to wash away their guilt for lying to the experimenter by being more helpful. Guilt made them more willing to engage in prosocial behavior. The opposite is also true. If people feel cleansed of guilt, they are less likely to engage in prosocial behavior (Zhong and Liljenquist, 2006; Xu et al., 2011). Previous research has not, however, measured whether guilt mediates the effect of cleansing on prosocial behavior. The present research fills this important gap in the literature.

### Washing the guilt away

Can washing one’s hands remove one’s guilt? Both Pilate and Lady Macbeth thought so, and they are not alone. Research has shown that people often feel less guilty after washing their hands (e.g., Zhong and Liljenquist, 2006; Nelissen and Zeelenberg, 2009; Bastian et al., 2011). Purity is the central notion of morality (Haidt and Joseph, 2008), and cleansing makes one more pure and clean (Lee and Schwarz, 2010). In baptisms and other religious rituals, water is used to wash away sin and make the person clean and pure.

Does one have to physically wash one’s own hands of guilt, or is it sufficient to watch others wash their hands? We suggest that watching others wash their hands might “wash away” at least some of the guilt. It has been suggested that embodiment plays an important role in helping the brain simulate experience, process information, form attitudes, arouse emotions, make decisions, and take actions (Niedenthal et al., 2005; Barsalou, 2008). According to embodied cognition theories (Gallese and Lakoff, 2005; Niedenthal, 2007; Barsalou, 2008; Meteyard et al., 2012), acting and simulating share the same brain substrates. When simulating an action, the brain (partially) reactivates the (original) action as well as any accompanying thoughts and feelings (Barsalou, 1999; Rubin, 2006; Niedenthal, 2007). Abstract concepts and emotions are grounded and “embodied” in our concrete experience and knowledge (Lakoff and Johnson, 1980, 1999). That is, abstract concepts and emotions can be comprehended and retrieved by concrete experience as well as by simulating the experience. It is thus plausible that the concepts of “cleanliness” and “purity” are embodied in bodily movements and everyday rituals such as erasing, rinsing, and washing.

Washing one’s hands is a “bottom-up” process grounded in authentic sensory and motor experiences that activates the concepts of “cleanliness” and “purity”. Watching others wash their hands is a “top-down” process in which the brain simulates comparable sensory and motor experiences. In both cases, guilt should be reduced due to either “bottom-up” reactivation of concepts of “cleanliness” and “purity” or “top-down” simulation of washing one’s hands. However, we propose that physically washing one’s hands should be more effective in reducing guilt than watching others wash their hands, for two reasons. First, the “bottom-up” experience of cleansing oneself is more perceptually convincing and vivid than the vicarious “top-down” simulation of cleansing oneself. Second, reliving or reenacting an experience only involves reactivation of part of the neurons engaged in the original experience (Damasio, 1989; Barsalou et al., 2003). This discrepancy in the amount of neurons between “bottom-up” reactivation and “top-down” simulation should cause the difference in their effect on reducing guilt. Therefore, watching others cleanse themselves might decrease one’s own guilty feelings to a lesser degree than washing one’s own hands. The present research therefore includes three experimental conditions: self-cleanliness, other-cleanliness, and no-cleanliness control.

### Overview of the present study

The present research expands past research in several important ways. First, it compares the effect of washing one’s own hands versus watching someone else wash their hands. Second, it includes a measure of prosocial behavior to measure the behavioral effects of washing one’s guilt away. Third, it tests whether guilt mediates the effect of cleanliness on prosocial behavior.

In the present study we first induced feelings of guilt by having participants recall and then write a detailed description about a past wrong they committed against a significant other (e.g., family member, close friend). Next, they were randomly assigned to one of three experimental conditions: (1) a *personal-cleanliness* condition in which they washed their own hands; (2) an *other-cleanliness* condition in which they watched a video of someone else wash their hands; or (3) a no-cleanliness *control* condition in which they watched a video of someone else typing. We measured feelings of guilt before and after the experimental manipulation. Participants were then told the study was over, and they were paid for their participation. The experimenter added, however, that if they wanted they could help a Ph.D. student complete her dissertation by taking some questionnaires home and returning them within 3 weeks in a prepaid envelope. The number of questionnaires returned was used to measure prosocial behavior. We predict that physical self-cleansing is more effective than a metaphorical concept of cleanness in decreasing guilt. But watching someone else wash his or her hands should “wash away” at least some of the guilt. Thus, we predicted the lowest levels of guilt and prosocial behavior among participants who washed their own hands, followed by participants who watched someone else wash their hands, followed by participants in the control condition who watched someone type with their hands. Furthermore, we expected guilt to mediate the effects of cleanliness on prosocial behavior such that the more guilty participants felt, the more helpful they would be.

## Method

### Ethics statement

Our study was approved by the ethical committee of Laboratoire Interuniversitaire de Psychologie (LIP) at the University of Grenoble, France. We also obtained consent from our participants.

### Participants

Participants were 65 adult patrons at a municipal library in France (30 women; 18–79 years old; *M*_age_ = 41.5, *SD*_age_ = 16.7) who were paid 10€ ($14) in exchange for their voluntary participation.

### Procedure

Participants were tested individually. They were told the researchers were studying the relationship between verbal memory, memory of body movements, and emotions. After giving their consent, participants were given 15 min to write a description about an event in which they had done something negative to someone important to them. This paradigm has been widely in past research used to induce guilt feelings in participants (e.g., Niedenthal et al., 1994; Smith et al., 2002; Lickel et al., 2005), and it is especially effective when the person they are writing about is someone important to them (Baumeister et al., 1994; Xu et al., 2011). Participants were told to write down the whole story, to include as many details as possible, and to describe exactly how it made them feel. Next, participants completed the 5-item (e.g., “I feel bad about something I have done”) guilt subscale of the State Guilt and Shame Scale (Marschall et al., 1994; Cronbach α = 0.87; *M* = 12.80, *SD* = 5.24) to measure their current feelings of guilt.

Next, participants completed a task that ostensibly measured memory of body movements. They were randomly assigned to three conditions: personal-cleanliness (*N* = 21), other-cleanliness (*N* = 22), or control (*N* = 22). In the *personal-cleanliness* condition, participants first memorized the numbers (from 1 to 14) on a paper for 1 min. Each number was paired with a finger, the palm, or the back of the left or right hand (i.e., 1 = thumb, 2 = index finger, 3 = middle finger, 4 = ring finger, 5 = little finger, 6 = palm, 7 = back of left hand; 8 = thumb, 9 = index finger, 10 = middle finger, 11 = ring finger, 12 = little finger, 13 = palm, 14 = back of right hand). The participant typed these numbers on a computer keyboard, and then wiped each finger, the palm, or the back of the appropriate hand in the order of the numbers with a wet white wipe for about 2 min. In the *other cleanliness* condition, participants watched a 2-min video of someone else doing the same thing as in the *personal-cleanliness* condition, and recalled the numbers in the appropriate order. In the *control* condition, participants also watched a 2-min video of a person typing numbers on a keyboard and recalled the numbers.

Next, participants again completed the State Guilt and Shame Scale (Marschall et al., 1994; Cronbach α = 0.81). Participants also completed the Positive and Negative Affect Schedule (PANAS; Watson et al., 1988; *M*_positive affect_ = 32.17, *SD*_positive affect_ = 6.32; *M*_negative affect_ = 19.09, *SD*_negative affect_ = 5.52) to test whether the effects of the manipulation were specific to guilt. This scale contains 10 negative items (*afraid*, *ashamed*, *distressed*, *guilty*, *hostile*, *irritable*, *jittery*, *nervous*, *scared*, and *upset*; Cronbach α = 0.80), and 10 positive items (*active*, *alert*, *attentive*, *determined*, *excited*, *enthusiastic*, *inspired*, *interested*, *proud*, and *strong*; Cronbach α = 0.82).

Participants were told that the study was over, but if they were willing to help a Ph.D. student complete her dissertation they could take some questionnaires (about local public transportation) home and mail them back within 3 weeks in a prepaid envelope. The experimenter recorded the number of questionnaires they took, and also how many they mailed back within 3 weeks.

## Results

### Preliminary analyses

Because age has been shown to positively correlate with guilt (Orth et al., 2010), we tested whether there were any main or interactive effects for age on any of the dependent variables (i.e., guilt, number of questionnaires taken, and number of questionnaires returned). No significant effects were found. Likewise, no significant main or interactive effects were found for participants’ sex, so the data from men and women were combined.

### Primary analyses

The means and standard deviations for all dependent variables are in Table 1.

**Table 1:** **Means of dependent variables as a function of condition**.

	Personal-cleanliness	Other-cleanliness	Control
Guilt (pre-test)	−0.26_a_ (0.83)	−0.023_a_ (1.02)	0.27_a_ (1.10)
Guilt (post-test)	−0.97_a_ (0.66)	0.080_b_ (0.65)	0.84_c_ (0.73)
Word “guilty”	2.10_a_ (0.70)	2.77_b_ (0.81)	3.32_c_ (1.00)
Positive affect	32.81_a_ (5.77)	32.86_a_ (6.49)	30.86_a_ (6.73)
Negative affect	14.76_a_ (4.38)	17.86_a_ (6.60)	16.05_a_ (5.38)
Number of questionnaires returned	0.24_a_ (0.54)	0.77_b_ (0.75)	2.36_c_ (2.08)
Proportion of questionnaires returned	14%_a_ (32%)	40%_b_ (40%)	52%_c_ (30%)

Note. Standard deviations are in parentheses. Negative affect was calculated without the item “guilty”. Means having the same subscript are not significantly different from each other at the 0.05 significance level.

#### Guilt

Guilt standardized scores were analyzed using a 3 (personal-cleanliness versus other-cleanliness versus control) × 2 (before versus after manipulation) mixed-model ANOVA. The predicted condition × time interaction was significant, *F*_(2,62)_ = 5.85, *p* = 0.004. As expected, guilt scores did not differ between conditions before the manipulation, *F*_(2,62)_ = 1.57, *p* = 0.22. Thus, random assignment to conditions was successful. After the manipulation, however, guilt scores differed across conditions, *F*_(2,62)_ = 10.75, *p* < 0.001. As expected, guilt scores were lower for participants in the *personal-cleanliness* condition than for participants in either the *other-cleanliness* condition (*d* = 1.05, *p* = 0.013) or the *control* condition (*d* = 1.81, *p* < 0.001). Guilt scores were also lower for participants in the *other-cleanliness* condition than for participants in the *control* condition (*d* = 0.76, *p* = 0.041).

#### Positive and negative affect

We also examined positive and negative affect to be sure our manipulation was specific to guilt. One-way ANOVA (personal-cleanliness versus other-cleanliness versus control) showed no impact of condition on positive affect, *F*_(2,62)_ = 0.71, *p* = 0.50. In addition, there was also no significant impact of condition on any of the other nine negative emotions (i.e., *afraid*, *ashamed*, *distressed*, *hostile*, *irritable*, *jittery*, *nervous*, *scared*, *upset*; *p*s > 0.10), or on all of the other nine negative emotions combined (Cronbach α = 0.82, *p* > 0.50).

There was, however, a significant impact of condition on the single item *guilty*, *F*_(2,62)_ = 10.75, *p* < 0.001, As expected, *guilty* scores were lower for participants in the *personal-cleanliness* condition than for participants in either the *other-cleanliness* condition (*d* = 0.69, *p* = 0.013) or the *control* condition (*d* = 1.24, *p* < 0.001). *Guilty* scores were also lower for participants in the *other-cleanliness* condition than for participants in the *control* condition (*d* = 0.55, *p* = 0.041).

#### Prosocial behavior

One-way ANOVA found a significant effect of condition on the number of questionnaires participants completed and returned to the researchers by post, *F*_(2,62)_ = 15.10, *p* < 0.001, As expected, participants in the *personal-cleanliness* condition returned fewer questionnaires than did participants in either the *other-cleanliness* condition (*d* = 0.34, *p* = 0.033) or the *control* condition (*d* = 1.00, *p* < 0.001). Participants in the *other-cleanliness* condition also returned fewer questionnaires than did participants in the control condition (*d* = 1.34, *p* < 0.001).

Because there was a significant correlation between the number of questionnaires taken and the number returned (*r* = 0.74, *p* < 0.001), we also computed the proportion of questionnaires taken that were completed and returned. The effect of condition was still significant, *F*_(2,62)_ = 6.73, *p* = 0.002. As expected, participants in the *personal-cleanliness* condition mailed back fewer questionnaires than did participants in either the *other-cleanliness* condition (*d* = 0.68, *p* = 0.019) or the *control* condition (*d* = 1.01, *p* < 0.001). The latter two conditions did not differ (*d* = 0.34, *p* > 0.24), although the effect-size estimate was not trivial. According to Cohen (1988), *d* = 0.2 is a “small” effect, *d* = 0.5 is a “medium” effect, and *d* = 0.8 is a “large” effect.

#### Mediating effect of guilt

**Figure 1:**
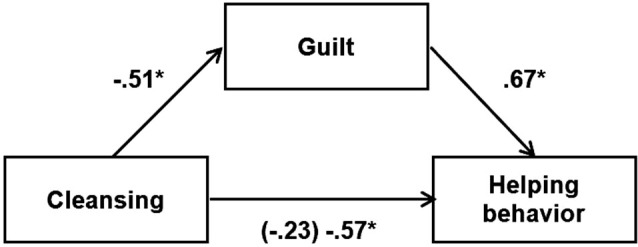
**Mediating effect of guilt on the relationship between cleansing and prosocial behavior**. *Note*. The standardized regression coefficient in parentheses was obtained from a model that included both cleansing and guilt as predictors of prosocial behavior. * *p* < 0.05.

We also used bootstrapping procedures (Preacher and Hayes, 2004) to test the mediating effects of guilt on the effect of condition (coded +1 = *personal-cleansing*, 0 = *other-cleansing*, −1 = *control*) on the number of questionnaires completed and returned. The results were presented in Figure 1, the standardized regression coefficient in parentheses was obtained from a model that included both cleansing and guilt as predictors of prosocial behavior. As can be seen in Figure 1, cleansing decreased guilt, and guilt, in turn, was positively related to prosocial behavior. The indirect effect of cleansing on prosocial behavior was significant, 95% confidence interval = −0.37 to −0.32, which excludes the value 0. Nearly identical results were obtained for the proportion of questionnaires returned (i.e., 95% confidence interval was −0.13 to −0.079, which also excludes the value 0).

## Discussion

The present study showed that one can indeed wash the guilt away by washing one’s hands, replicating previous studies and supporting current embodiment theories that argue that abstract concepts (in our case cleanliness and purity) are bodily embodied and reinstantiated by sensory and motor inputs. The present research, however, does not simply replicate previous research—it extends it in three important ways. First, it compared the effect of washing one’s own hands versus watching someone else wash their hands. This comparison showed that although washing someone else wash their hands can cleanse some guilt away, it is not as effective as washing one’s own hands. Thus, vicarious experience of cleanliness is not as effective as the action of cleansing (i.e., the personal embodiment of cleansing). However, watching someone else wash his or her hands did have an effect on reducing guilt compared with the control condition. Our findings suggest that while watching another person wash his or her hands, the brain simulates the comparable sensory and motor experience so that it induces vicarious feelings of “cleanliness” and primes the concepts of “cleanliness” and “purity”, which counteracts and reduces feelings of guilt and its consequent effect on promoting prosociality. However “top-down” simulation might not be as vivid and convincing as “bottom-up” reactivation, perhaps due to less activated neurons in visual and motor modalities. It is also plausible that the concepts of “cleanliness” and “purity” are more likely to be embodied in tactile and olfactory modalities rather than in the visual modality (e.g., Schnall et al., 2008). Our findings contribute to embodiment theories in that they showed the effect of vicarious cleansing on reducing guilt, and that vicarious cleansing may be less effective than personal embodiment of cleansing.

Second, the present research included a measure of prosocial behavior to measure the behavioral effects of washing one’s guilt away. Participants could help a Ph.D. student complete her dissertation simply by completing some questionnaires, in the comfort of their own home, and within a lengthy time period (i.e., 3 weeks). As expected, participants who washed their own hands completed the fewest number questionnaires within the 3-week period. It is remarkable that the effects of washing one’s hands can last up to 3 weeks. The difference in proportion of questionnaires returned between the other-cleanliness and personal-cleanliness condition suggests that “bottom-up” reactivation might have longer effect than “top-down” simulation on activating concepts of “cleanliness” and “purity”. Again, this might be attributed to fewer neurons involved in the embodying process than in the actual experience.

Third, the present study explains why cleansing decreases prosocial behavior. Our mediation analysis showed that cleansing, especially personal-cleansing, reduced guilt. The less guilty participants felt, in turn, the less likely they were to help the Ph.D. student complete her dissertation. No previous study has included all the three elements (i.e., cleansing, guilt, and prosocial behavior).

This study, like most studies, raises questions as well as answers them. It’s still not clear whether and how guilt *per se* is embodied somewhere in the brain’s multi-modal system. Does guilt share the same modalities with concepts such as “cleanliness” and “purity?” Does it demand more interoceptive stimuli inputs? How do self-representations fit into the framework of embodiment? Future research should address these questions. In addition, future research should apply embodied cognition theories to self-conscious emotions (pride, guilt, embarrassment, shame, etc.) whose phylogeny is generally inferred based on reasoning independent of perceptual modalities.

In summary, Pilate and Lady Macbeth probably did feel less guilty after washing their hands, much like the participants in our study. Washing one’s hand can wash the guilt away. Unfortunately, washing one’s hands of guilt can also reduce prosocial behavior. Although washing one’s hands is good for hygiene, it is bad for social relationships.

## Author contributions

Hanyi Xu and Laurent Bègue designed the experiment. Hanyi Xu performed the study. Hanyi Xu and Brad J. Bushman conducted the data analyses. The experiment was carried out in the lab of Laurent Bègue. Hanyi Xu, Laurent Bègue, and Brad J. Bushman wrote the main manuscript text. All authors reviewed the manuscript.

## Conflict of interest statement

The authors declare that the research was conducted in the absence of any commercial or financial relationships that could be construed as a potential conflict of interest.
